# MicroRNA-7 Inhibits Rotavirus Replication by Targeting Viral NSP5 In Vivo and In Vitro

**DOI:** 10.3390/v12020209

**Published:** 2020-02-13

**Authors:** Yan Zhou, Linlin Chen, Jing Du, Xiaoqing Hu, Yuping Xie, Jinyuan Wu, Xiaochen Lin, Na Yin, Maosheng Sun, Hongjun Li

**Affiliations:** Institute of Medical Biology, Chinese Academy of Medical Science & Peking Union Medical College, Yunnan Key Laboratory of Vaccine Research and Development on severe Infectious Disease, Kunming 650118, China; yanyan_850@163.com (Y.Z.); chenll1128@163.com (L.C.); dujing0217@hotmail.com (J.D.); huxiaoqing@imbcams.com.cn (X.H.); Xieyuping@imbcams.com.cn (Y.X.); wujinyuan@imbcams.com.cn (J.W.); linxiaochen@imbcams.com.cn (X.L.); yinna@imbcams.com.cn (N.Y.); maoshs@imbcams.com.cn (M.S.)

**Keywords:** rotavirus, microRNA 7, non-structural protein 5, gene replication, anti-viral

## Abstract

Rotavirus (RV) is the major causes of severe diarrhea in infants and young children under five years of age. There are no effective drugs for the treatment of rotavirus in addition to preventive live attenuated vaccine. Recent evidence demonstrates that microRNAs (miRNAs) can affect RNA virus replication. However, the antiviral effect of miRNAs during rotavirus replication are largely unknown. Here, we determined that miR-7 is upregulated during RV replication and that it targets the RV NSP5 (Nonstructural protein 5). Results suggested that miR-7 affected viroplasm formation and inhibited RV replication by down-regulating RV NSP5 expression. Up-regulation of miR-7 expression is a common regulation method of different G-type RV-infected host cells. Then, we further revealed the antiviral effect of miR-7 in diarrhea suckling mice model. MiR-7 is able to inhibit rotavirus replication in vitro and in vivo. These data provide that understanding the role of cellular miR-7 during rotaviral replication may help in the identification of novel therapeutic small RNA molecule drug for anti-rotavirus.

## 1. Introduction

Rotavirus (RV) is one of the most common causes of severe diarrhea in infants and young children under five years of age. Although live attenuated vaccines are available to protect against rotavirus infection, there are still approximately 125 million episodes of rotavirus gastroenteritis every year, resulting in approximately 200,000 child fatalities [1]. To date, there are no effective drugs for the treatment of rotavirus in addition to the preventative live vaccine [2]. The mechanisms of rotavirus infection and proliferation, especially the regulatory role of small RNA molecules, remains to be elucidated.

Rotavirus belongs to the family Reoviridae. It is a non-enveloped virus and possesses icosahedral capsids. The double-stranded RNA genome of rotavirus consists of 11 distinct segments encoding six structural proteins (VP1 to VP4, VP6, and VP7) and six non-structural proteins (NSP1 to NSP6) [3]. The genome of rotavirus is encapsulated by three layers of constructive proteins. The innermost core layer is composed of VP2 and encases the viral genome, RNA polymerase VP1, and capping enzyme VP3. The middle layer is composed of VP6, which along with VP2, forms the double-layered particles (DLPs). The outermost layer is composed of VP4 and VP7 and contains the main epitope region of rotavirus. Following infection, loss of the VP4 and VP7 outer layers yields the transcriptionally active DLP, activating gene transcription and viral protein synthesis. Replication of the rotavirus genome and assembly of the DLPs are primarily accomplished within the viroplasm, which is formed by the non-structural viral proteins NSP2 and NSP5 [4].

NSP5, which is encoded by genome segment 11, is a phosphorylated and serine- and threonine-rich (25% of total amino acids) protein, which often presents as a dimer [5,6]. After translational modifications, the molecular weight of the NSP5 isoforms range from 22 to 35 kD [7]. Under the regulation of NSP4-mediated Ca^2+^ [8], serine- and threonine-rich NSP5 converts between hypo-phosphorylated and hyper-phosphorylated isoforms [9,10]. By interacting with NSP2 [11], NSP5 participates in the formation of the viroplasm [12,13] and plays a key role in rotavirus replication [14,15]. In infected cells, replication of the rotavirus genome occurs in the viroplasm, which protects against natural immune surveillance. It has been reported that blocking the production of NSP5 by small interfering RNA (siRNA) can inhibit viral replication, decrease structural and non-structural protein production, reduce the synthesis of viral genome dsRNA, and decrease the production of infectious particles [16]. The phosphorylation status of NSP5 regulates rotavirus mRNA formation [17]; therefore, NSP5 is critical to the replication and infectivity of rotavirus progeny.

MicroRNAs (miRNAs) are non-coding small RNA molecules, which contain about 22 nucleotides [18,19]. miRNAs regulate the expression of more than one-third of eukaryotic genes and are involved in almost all biological processes, such as cell differentiation, proliferation, apoptosis, and metabolism, either by inhibiting translation or by causing mRNA degradation at the post-transcriptional level [20,21,22,23]. Throughout evolution, the host has developed antiviral strategies via host–virus interplay [24,25,26]. During viral infection, the host cell inhibits viral proliferation and infection by regulating its own microRNAs to either target viral genes or its own signaling pathway genes [27,28]. MicroRNA-7 (miR-7) is a highly conserved miRNA among different species. In humans, MIR-7 is encoded by three different genomic loci: 9q21, 15q26, and 19q13. The three different primary *MIR-7* products (termed as pri-miR-7) are then processed into the mature MIR-7 sequence, which is comprised of 23 nucleotides. MiR-7 has been studied extensively in human organ/tissue development (including brain, pancreas, and thymus) [29,30,31], tumor biology (growth, migration, and immune escape) [32,33,34], and pathogenesis of diabetes [30,35]. However, the mechanism of miR-7 in antiviral defense has not been well studied. It has been demonstrated that a specific siRNA plays a critical role in inhibiting poliovirus (PV) replication [36,37]. The siRNA was able to not only effectively inhibit viral replication and reduce PV titers, but also upregulate miR-7 expression in host cells, which in turn, led to enhanced inhibition of PV infection [36]. It has also been reported that miR-7 targeted and inhibited the expression of the immune-related factor Myd88 in crabs and was able to influence the replication of the white spot syndrome virus (WSSV) [38]. However, there are very few reports regarding the role of miR-7 in regulating rotavirus replication. In this study, we explored the role of miR-7 in rotavirus replication, its mechanism of antiviral defense, and its anti-rotavirus treatment potential.

In a previous study, we isolated the wild-type rotavirus ZTR-68 strain and allowed it to infect MA104 cells. We then performed deep RNA sequencing and analyzed the microRNA expression profile [39]. We found that the expression levels of 40 microRNAs were altered during rotavirus infection. Among these microRNAs, the expression level of *MIR-7* was significantly upregulated during rotavirus infection, and we further confirmed this upregulation by quantitative real-time polymerase chain reaction (qRT-PCR). In the present study, we find that MIR-7 was able to inhibit rotavirus replication both in vitro and in vivo, which was achieved by targeting the rotavirus gene that encodes NSP5 and influencing viroplasm formation and viral genome replication.

## 2. Materials and Methods

### 2.1. Ethics Statement

Collection and use of human stool specimens were approved by the Ethics Committee of the Institute of Medical Biology (Yishenglunzi (2011) 16), and was provided following children’s stool samples with written informed consent from the next of kin or guardians. We received Institutional Review Board approval to use the samples and all samples were anonymized. The Yunnan Provincial Experimental Animal Management Association (approval number: SCXK (Dian) K2017-0002) and the Experimental Animal Ethics Committee of the Institute approved the animal research (approval number: DWSP201802001).

### 2.2. Cells and Virus

Fetal rhesus monkey kidney cells (MA104) were cultured in MEM (hyclone) medium containing 10% (vol/vol) newborn bovine serum (Min Hai, China) and 1% (vol/vol) penicillin and streptomycin. HEK293 cells were maintained in DMEM (Invitrogen) supplemented with 10% (vol/vol) fetal bovine serum (FBS) (Gibco, Grand Island, NY, USA). HT-29 cells were maintained in McCoy’s 5A medium supplemented with 10% FBS. Caco-2 cells were maintained in DMEM supplemented with 10% FBS, 1% NEAA (Gibco, Grand Island, NY, USA), and 1% sodium pyruvate. Cells were passaged when confluence reached 98%. The wild rotavirus strain ZTR-68 (G1P [8]) was isolated from a fecal sample from a 5-month-old infant in Yunnan province, China in 2010 [40]. We adaptive cultured rotavirus strain ZTR-68 in MA104 cells and Vero cells. Rotavirus strain ZTR-68 cultured in MA104 cells named M-ZTR-68 and that cultured in Vero cells named V-ZTR-68. Simian rotavirus strain SA11 (G3P [2]), human rotavirus strain S2 (G2P [4]) and porcine rotavirus strain Gottfried (G4P [8]) were kept in our lab. The wild rotavirus strain ZTR-18 (G9P [8]) was isolated from a fecal sample from a infant in Yunnan province, China in 2013.

### 2.3. MA104 Cells Transfection, Viral RNA and Protein Detection

MIR-7 mimics and inhibitors were synthesized and transfected into MA104 cells. Briefly, MA104 cells were seeded at 6 × 10^4^ cells/well in a 6-well plate 24 h prior to transfection with 100 nmol miR-7 mimic and inhibitor. Twenty-four hours after transfection, MA104 cells were infected with rotavirus strain at 0.5 MOI. At 36 h post infection (hpi), total RNA was extracted using TRIzol^TM^ Reagent (15596026, ambion, USA) for RT-PCR assay. Meanwhile, viral RNA was extracted using GeneJET Viral DNA and RNA Purification Kit (#K0821, Thermo Scitific, USA). Then, the viral genome was detected by RNA polyacrylamide gel electrophoresis (PAGE) and silver dye.

Western blot analysis of NSP5 in miR-7-upregulated and downregulated MA104 cells that were transfected with 100 nmol miR-7 mimic and inhibitor for 24 h before RV infection. The lysates were analyzed by Western blot using rabbit anti-NSP5(0.1 μg/mL, Genscript, Nanjing, China) and mouse anti-β-actin(1:1000,#3700, Cell signaling, MA, USA) at 12, 24, 36 and 48 hpi.

### 2.4. Prediction of Binding Site to miR-7 by miRanda Software

The target site of miR-7 in different genotype rotaviruses were predicted by the Miranda algorithm following the rules of miRNA-3’ Untranslated Region (UTR) sequence matching and energy stability assessment in the miRNA seed sequences [41,42]. The algorithm uses dynamic programming to search stable duplex regions of miRNAs complementary to the 3’UTR of target genes. The following threshold parameters were used in the prediction process: S ≥120 and ΔG ≤−20 kcal/mol, where S refers to matching region single-residue-pair match scores and ΔG is the free energy of formation of double-stranded.

### 2.5. Luciferase Assay

Cells cultured in a 24-well plate were washed once with phosphate buffer saline (PBS), and then 100 μL lysis buffer (Promega, USA) was added to each well. We then mixed 50 μL of each sample with luciferase assay substrate (Promega, USA). Luciferase activity was measured for 10 s using a luminometer (Turner Designs TD-20/20). Assays were performed in triplicate, and luciferase activity is presented as the mean ± standard deviation (SD).

### 2.6. Immunofluorescence Staining

MA104 cells were transfected with 100 nmol miR-7 mimic, inhibitor and negative oligonucleotide for 24h before RV infection. Then, the cells were infected with rotavirus at a multiplicity of infection (MOI) of 0.5. Cells were assayed for infectious rotavirus by fluorescent assay using rabbit anti rotaviral VP7 antibody (Genscript, Nanjing, China) at 24 hpi. Cells were fixed with 2% paraformaldehyde containing 0.2% Triton X-100 for 30 min at 4 °C, then fixed with pre-cooled 98% methanol for 10 min at 4 °C. After washing with ice-cold PBS, samples were blocked with 2% bovine serum albumin (Sigma) in PBS for 1 h at 37 °C. The slides containing cells were incubated with antibodies, such as rabbit anti-VP7 (0.1 μg/mL, Genscript, Nanjing, China) and rabbit anti- NSP5 (0.1 µg/mL, Genscript, Nanjing, China) for 1.5 h at 37 °C. After incubation, cells were washed with PBS and incubated with goat anti-rabbit IgG-DyLight™ 594 (0.5 μg/mL, Jackson ImmunoResearch, West Grove, PA, USA) for 1 h at 37 °C. The slides were then washed three times with PBS and covered with a cover slip, followed by staining with 4, 6-diamidino-2- phenylindole dihydrochloride (Sigma, USA). The negative controls were performed in parallel. Samples were analyzed using a fluorescence microscope (Nikon, Tokyo, Japan). The average numbers of positive cells in at least 5 fields were quantified in different group.

### 2.7. Virus Titer Detection

Viral titer plaque assay was conducted as previously described [43]. Briefly, MA104 cells were grown in six-well tissue culture plates for 3 to 5 days, until they formed tightly packed monolayers. Rotavirus were activated using 20 μg/mL trypsin and 800 μg/mL CaCl_2_ in a 37 °C water bath for 1 h. The activated virus was used to create a dilution series ranging from 10^−2^ to 10^−7^. 1 mL of a virus dilution was added to each well at 37 °C for 1 h adsorption. Then the inoculum was removed, and the cells were washed using 2 mL pre-warmed, serum-free MEM medium. The medium was removed, and the cells were gently overlaid with 3 mL/well of a 3% agarose/medium mixture containing 5 μg/mL trypsin. The plates were inverted cultured at 37 °C incubator for 3 to 4 days. A mixture of 3% agarose in serum-free MEM medium with 50 μg/mL neutral red was prepared, and 2 mL/well of this mixture was overlaid on top of the first agarose/medium overlay. The six-well plates were kept in a stainless-steel box to protect them from light and to allow the agarose to harden. Finally, the individual plaques were counted using a light box.

### 2.8. Viral Load Detection and Quantitative Real-Time Polymerase Chain Reaction (qRT-PCR) Assay

Viral load was detected by qRT-PCR using NSP3 probe primer [44]. Total RNA was extracted from cells and small intestine using TRIZOL Reagent (15596026, ambion, USA) according to the manufacturer’s specifications. The sequences for the primers were as follows: up primer, 5′-GTTGTCATCTATGCATAACCCTC-3′; down primer, 5′-ACATAACGCCCCTATA GCCA-3′; 6-carboxyfluorescein (FAM)-labeled probe, 5′-ATGAGCACAATAGTTAAAAGCTAACACTGTCAA-3′. Viral load was measured by detecting expression of NSP3 gene. A standard reference curve was obtained by measurement of standard virus RNA (10^6.0^ copies/mL). According to the standard reference curve, the viral load was quantified in each sample. RT-PCR assays were performed using the Trans Script II Probe One-Step qRT-PCR SuperMix (TRANS, AQ321-01) in Real-Time System (CFX96, BIO-RAD, USA). The reaction system included 5μL RNA template, up primer at 20nm, down primer at 20 nm, FAM-labeled probe at 20 nm and E-mix in a 20 μL total reaction volume. Reactions were incubated in a 8-well optical plate (BioRad, USA) at 50 °C for 5 min, 94 °C for 30 s, followed by 40 cycles of 94 °C for 5 s and 60 °C for 30 s.

### 2.9. Observation of Viroplasm by Transmission Electron Microscopy (TEM)

Viroplasm observations was conducted as previously described [43]. Briefly, infected MA104 cells were washed once with PBS at 12 hpi and collected by centrifugation at 1000× *g* for 5 min at 4 °C. The precipitated cells were fixed in 2.5% electron microscopy-grade glutaraldehyde, rinsed with 0.2 M sodium cacodylate buffer, and then fixed in 1% osmium tetroxide (Nanjing Zhongjingkeyi Technology, Co., Ltd., Nanjing, China) for 2 h. The cells were rinsed with 0.2 M sodium cacodylate buffer three times and overnight. The cells were then dehydrated through a graded series (30%, 50%, 70% and 90%) of ethanol dilutions, 90% acetone and pure acetone, then processed for Epon™ embedding (Sigma-Aldrich; Merck Millipore). Ultra-thin sections (60 nm) were stained with uranyl acetate and lead citrate (Nanjing Zhongjingkeyi Technology, Co., Ltd.) and were then observed using H-7650 electron microscope (HITACHI, Ltd., Tokyo, Japan).

### 2.10. Animal Inoculation and Virus Challenge

CY5 labeling reagents including the agonist miR-7 agomir, antagonist miR-7 antagomir, and negative control (negative oligonucleotide control) were synthesized and modified by Ruibo Biology company (Guangzhou, China) for animal experiments. Six-to 4-day-old Balb/c infant mice were divided into groups of three including negative control group, miR-7 agomir group and miR-7 antagomir group. Twenty-four hours before virus infection, each three groups of mice were respectively given by an intragastric inoculation of 10 nmol negative oligonucleotide, 10 nmol miR-7 agomir and 20 nmol miR-7 antagomir at days 4 of life. Then, the 5-day-old mice were challenged using oral needle by an intragastric inoculation of 10 infectious dose 50 (ID50) (10^5.0^ plaque-forming units, PFU) rotavirus. Virus challenge was performed as described previously [45,46]. Mock infected mice were inoculated with equal volume of medium from uninfected MA104 cells. Inoculated mice were housed with mother mice and were examined per 12 h for diarrhea by gentle palpation of the abdomen. Inoculated mice were evaluated for diarrhea (0 to 4), no fecal discharge recorded score 0, brown molded stool recorded score 1 and brown soft stool recorded score 2 and soft yellow stool recorded scores 3, yellow watery stool recorded score 4, perianal stool contamination recorded score 4. 1 was considered no diarrhea; 2 was considered common diarrhea; 3 was considered severe diarrhea; 4 was considered very severe diarrhea. Mice with a score >2 were identified as having diarrhea. The suckling mice were euthanized at 12, 24, 48, 72 and 96 hpi, and intestinal tissues were harvested. Total RNA was extracted after the tissue was homogenized by grinding the tissue while frozen using TRIZOL Reagent (15596026, ambion, USA) for rotavirus copy number detection by qRT-PCR.

### 2.11. Histopathological and Jejunum Section Immunofluorescence

The small intestine of the infected and euthanatized infant mice at 48 h post infection (hpi) were collected and checked histopathological changes. The small intestine sections were fixed in 10% (*v/v*) formalin in PBS for at least 48 h, then dehydrated in 70% (*v/v*) graded ethanol series and embedded in paraffin before being sectioned at the 4.0 μm thickness for further hematoxylin and eosin (H&E) staining. Histopathological observation of the small intestine was carried out under a light microscope. The jejunum paraffin sections of infected mice were assayed using Goat anti-rotavirus antibody (1:1000, made by our lab) and mouse anti-keratin 18 (red fluorescence represents keratin 18, #4548, Cell signaling, MA, USA). After incubation, slides were washed with PBS and incubated with donkey anti-Goat IgG-FITC (0.5 μg/mL, Jackson ImmunoResearch, West Grove, PA, USA) and rabbit anti-mouse IgG-DyLight™ 594 (0.5 μg/mL, Jackson ImmunoResearch, West Grove, PA, USA) for 1 h at 37 °C. The slides were then washed three times with PBS and covered with a cover slip, followed by staining with 4, 6-diamidino-2- phenylindole dihydrochloride (Sigma, USA). Samples were analyzed using a fluorescence microscope (Nikon, Tokyo, Japan).

### 2.12. Statistical Analysis

Between-group differences were statistically analyzed using a two-tailed Student’s *t*-test or Tukey’s multiple comparison test using GraphPad Prism (Version 5.01). *p* values of <0.05 were considered significant.

## 3. Results

### 3.1. Detection of MIR-7 Expression during Rotavirus Replication

**Figure 1:**
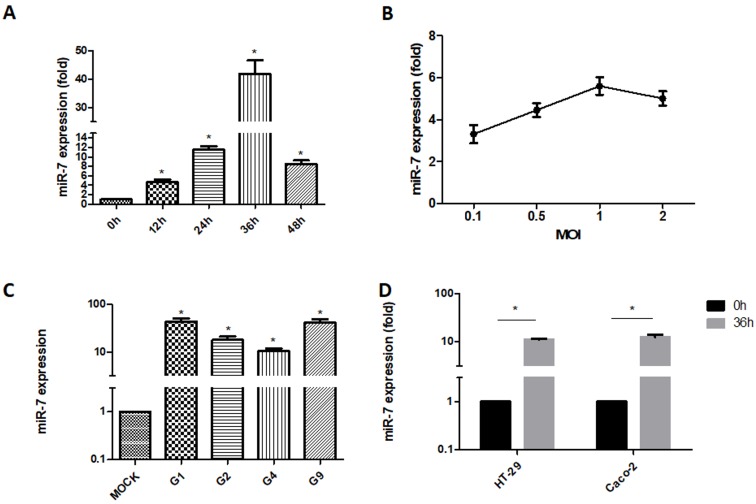
Expression detection of MiR-7 using real-time polymerase chain reaction (RT-PCR). Control, mock-infection. (**A**) MiR-7 expression analysis at 0, 12, 24, 36 and 48hpi (hours post infection) that were infected with the wild-type rotavirus (RV) strain ZTR-68; (**B**) MiR-7 expression analysis in different multiplicity of infection (MOI) at 36 hpi; (**C**) MiR-7 expression levels in RVs of other genotypes, including G1(ZTR-68), G2(S2), G4 (Gottfried), and G9(ZTR-18); (**D**) MiR-7 expression level in HT-29 and Caco2 cells that were infected with the wild-type RV strain ZTR-68. Data shown represent one of three separate experiments performed in quadruplicate. ‘Fold’ stands for the miR-7 expression is increased in comparison to mock group. * *p* ≤ 0.01 compared with Control cells. Error bars represent SD.

Total RNA was extracted from MA104 cells infected with ZTR-68 (MOI = 0.1) at 12, 24, 36, and 48 h post-infection(hpi). qRT-PCR results showed that the expression of miR-7 continued to increase until it peaked at 36 hpi (Figure 1A). We used G1 type ZTR-68 rotavirus to infect MA104 cells with different MOIs (0.1, 0.5, 1, and 2). We then extracted total RNA from the cells at 24 h post-infection and measured the expression of miR-7 by qRT-PCR. Our results showed that MiR-7 expression increased as the MOI of rotavirus infection increased (Figure 1B). The miR-7 expression was upregulated in MA104 cells infected with different rotavirus strains (at a MOI of 0.5), including G1, G2, G4 and G9, and was most notable in cells infected with the G1 strain (Figure 1C). Caco-2 and HT-29 cells were infected with the ZTR-68 virus (MOI = 0.5), and total RNA was extracted. The expression of miR-7 was measured by qRT-PCR at 36 hpi. The qRT-PCR results indicated that rotavirus infection caused a significant upregulation of miR-7 expression in these cells (Figure 1D).

### 3.2. The Role of MIR-7 in Rotavirus Replication

**Figure 2:**
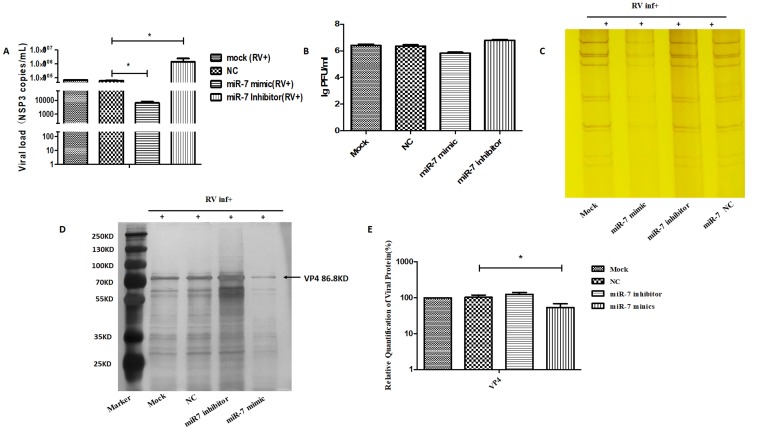
The role of miR-7 during rotavirus replication. MA104 cells were transfected with 100 nmol negative control oligonucleotides (NC), miR-7 mimic and inhibitor for 24 h before RV infection. (**A**) Viral gene (NSP3) copies detection by RT-PCR. At 36 hpi, RNA of viruses was extracted for RT-PCR assay. Data are expressed as the mean ± standard deviation (SD) *n* = 3, * *p* < 0.01. (**B**) Viral titer of the yield of rotavirus were detected by plaque assay. At 72 hpi, viruses were harvested for plaque assay; (**C**) Viral genome detection by PAGE. At 36 hpi, RNA of viruses was extracted for PAGE detection and silver dye. (**D**) Viral protein detection by SDS-PAGE. At 72 hpi, viruses were harvested for sodium dodecyl sulfate polyacrylamide gel electrophoresis (SDS-PAGE). (**E**) Quantification of the viral VP4 protein detected in Figure 2D by gray value comparison.

MIR-7 mimics and inhibitors were synthesized and transfected into host cells (MA104 cells). Twenty-four hours after transfection, the host cells were infected with rotavirus. The intracellular viral RNA was extracted at 36 hpi for viral RNA copies and RNA PAGE detection. The gene copy number of progeny virus was measured by a probe that detects *NSP3* in the viral genome. The copy number of *NSP3* increased faster in host cells with miR-7 downregulation than in cells with miR-7 upregulation (Figure 2A). Compared with the control cells, the gene copy number of progeny virus was lower in cells that had increased miR-7 expression. RNA PAGE results showed that the genomic pattern of harvested virus was the same, which was a 4-2-3-2 pattern (Figure 2C). Compared with the control and negative control (negative oligonucleotide control, NC) group, the amount of rotavirus RNA was significantly lower in the group with upregulated expression of *MIR*-*7* (Figure 2C). These results suggest that miR-7 inhibits rotavirus genome replication.

**Figure 3:**
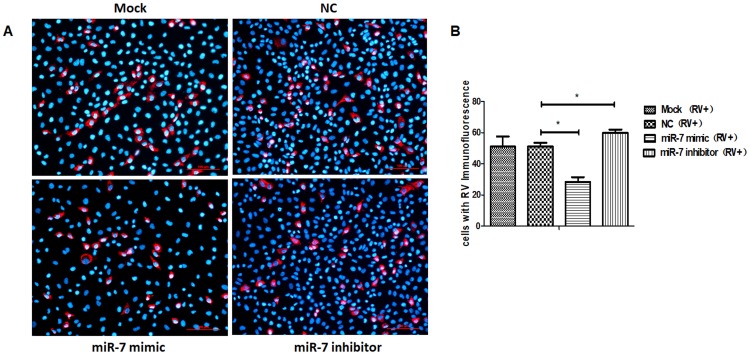
Immunofluorescence assay of Rotaviral VP7. Red represents VP7 signal, blue represents 4’,6-diamidino-2-phenylindole (DAPI) signal. (**A**) VP7 expression detection in miR-7-upregulated and downregulated MA104 cells that were transfected with 100 nmol miR-7 mimic and inhibitor for 24 h before RV infection. At 24 hpi, cells were assayed for infectious rotavirus by fluorescent assay using rabbit anti rotaviral VP7 antibody. (**B**) Quantification of VP7 signal positive cells, statistically significant differences. The average numbers of positive cells in at least 5 fields were quantified in different group. (* *p* < 0.01).

We collected the progeny virus of rotavirus-infected cells and control cells at 72 hpi for the viral titer analysis by plaque assay and protein detection by SDS-PAGE. The results showed that, compared with the control group, the proliferation of rotavirus progeny was lower in cells expressing an upregulation in miR-7 expression and was higher in cells demonstrating a downregulation in the expression of miR-7(Figure 2B). Compared with the control cells, the total protein was significantly lower in cells that had increased miR-7 expression (Figure 2D). Take the expression of viral VP4 protein as a representative for statistics (Figure 2E). Meanwhile, the expression of rotaviral VP7 protein was detected 24 hpi by immunofluorescence in MA104 cells that had either an increase or decrease in miR-7. The results also indicated that miR-7 is able to inhibit the expression of rotavirus proteins (Figure 3A,B).

### 3.3. MIR-7 Regulates Rotavirus Gene Replication by Targeting the *NSP5* Gene

**Figure 4:**
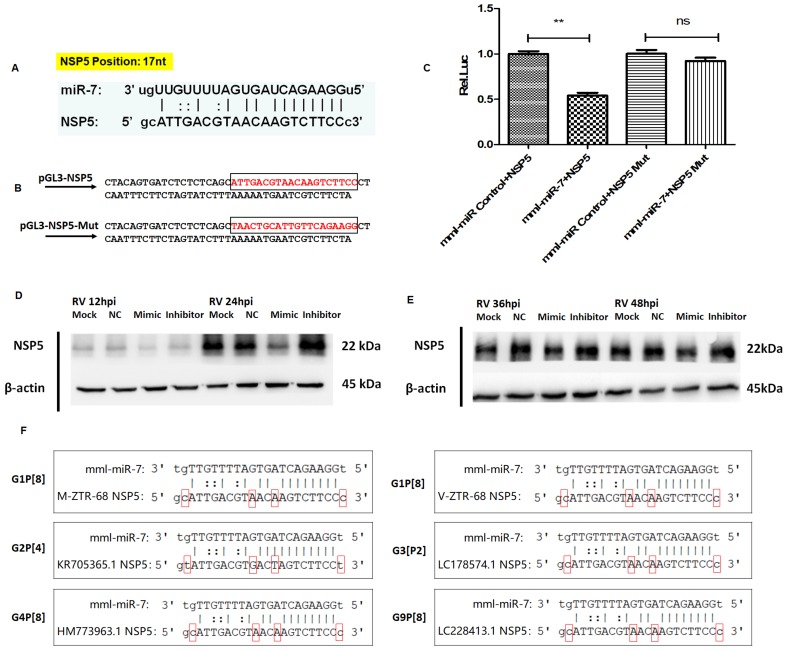
Interaction between miR-7 and NSP5. (**A**) Identification of miR-7 target location within *NSP5* gene. The target region of NSP5 were predicted with miRanda v3.3a algorithm. (**B**) pGL-TK plasmid constructs of the wild-type and mutated region of NSP5. The target site sequence complementary to the seed region of miR-7 are marked. (**C**) Analysis of luciferase activity. HEK293 cells were transfected with miRNA mimics or negative control oligonucleotides (miR control), pRL-TK and firefly luciferase reporter plasmid containing putative miRNA targeting sequences or mutant target sequence of *NSP5*. The results shown are mean ±SD of triplicate cultures, representative of three experiments (** *p* < 0.05); (**D**,**E**) Western blot analysis of NSP5 in miR-7-upregulated and downregulated MA104 cells that were transfected with 100 nmol miR-7 mimic and inhibitor for 24 h before RV infection. The lysates were analyzed by Western blot using antibodies against NSP5 and β-actin at 12, 24, 36 and 48 hpi. (**F**) Identification of miR-7 target location within *NSP5* gene of different genotype rotavirus including G1P [8], G2P [4], G3P [2], G4P [8] and G9P [8]. M-ZTR-68 represents ZTR-68 rotavirus adapted on MA104 cells. V-ZTR-68 represents ZTR-68 rotavirus adapted on Vero cells. The target region of *NSP5* were predicted with the miRandav3.3a algorithm.

After determining the role of miR-7 in rotavirus proliferation, we used miRanda software to analyze and identify the target gene of miR-7 among 11 rotavirus genes. The results suggested that miR-7 targets the *NSP5* gene (17 bp to 36 bp) in rotavirus strain ZTR-68 (Figure 4A). To identify the versatility of this binding site in different G-type rotaviruses, NSP5 sequences of G1P [8], G2P [4], G3P [2], G4P [8] and G9P [8] genotype human rotavirus were chosen for prediction of binding ability to miR-7 by miRanda software. The results suggested that miR-7 targets the NSP5 gene of different genotype RV at same genetic locus (Figure 4F). Although this site has a difference of 2–3 bases, the binding site is not affected. MiR-7 binds to the NSP5 gene of different G-type rotaviruses (Figure 4F).

**Figure 5:**
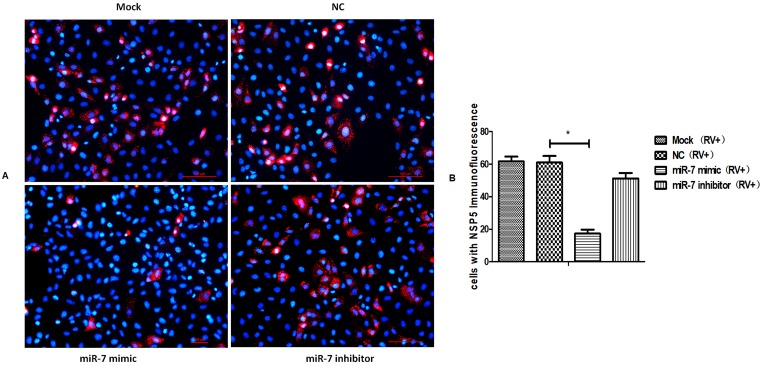
Immunofluorescence assay of Rotaviral NSP5. Red represents NSP5 signal, blue represents DAPI signal. (**A**) NSP5 expression detection in miR-7-upregulated and downregulated MA104 cells that were transfected with 100 nmol miR-7 mimic and inhibitor for 16 h before RV infection. At 16 hpi, cells were assayed for infectious rotavirus by fluorescent assay using rabbit antibody anti rotaviral NSP5. (**B**) Quantification of NSP5 signal positive cells, statistically significant differences. The average numbers of positive cells in at least 5 fields were quantified in different group. (* *p* < 0.01).

We further confirmed these results using a dual luciferase reporter system. The target sequence (NC) and the mutant sequence (NSP5 Mut) of the target gene *NSP5* were cloned into a luciferase expression plasmid (Figure 4B) and then co-transfected them into HEK293 cells with miR-7 mimics or control microRNA sequences. The results showed that miR-7 had a significant regulatory effect on the expression of luciferase when co-transfected with the target *NSP5* sequence, while the effect was not shown when co-transfected with the mutant miR-7-binding sequence of NSP5. This result indicated that miR-7 regulates the expression of NSP5 through this binding site (Figure 4C). To further confirm that miR-7 can regulate the expression of the *NSP5* gene, we transfected miR-7 mimics and inhibitors into MA104 cells and 24 h later infected the cells with rotavirus. We collected total cellular proteins at different time (12h, 24h, 36h and 48h) post-infection (hpi). The expression of the NSP5 protein after infection was measured by Western blot. It was found that the expression of NSP5 protein in host cells infected with RV was regulated by miR-7. And the inhibition is the most effective strongest at 24 hpi (Figure 4D,E). Rotavirus NSP5 protein was detected 16 h post-infection by immunofluorescence in MA104 cells that had either an increase or decrease in miR-7. The expression of NSP5 protein, shown as the intracellular dot aggregation, was significantly lower (Figure 5). It further demonstrated that *NSP5* is a target gene of miR-7.

**Figure 6:**
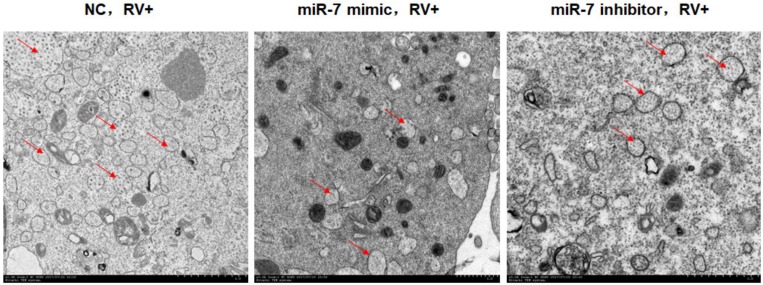
Viroplasm observation by transmission electron microscopy. RV-infected cells were harvested at 12 hpi and observed using H-7650 electron microscope. Arrowheads represent rotaviral viroplasm in cells. The scale bar of magnification of Figure 6 is 1 µm (10 equal parts).

Since NSP5 is the main protein involved in rotavirus viroplasm formation, we used transmission electron microscopy to observe the number and structure of viroplasms in MA104 cells at 12 hpi. Compared with the NC group, cells with miR-7 upregulation showed a significant decrease in the number of viroplasm, and the structure of viroplasm was incomplete. The difference between the NC cells and cells with downregulated miR-7 was not significant (Figure 6).

### 3.4. The Role of MIR-7 in a Rotavirus Diarrhea Model

In order to further study the antiviral effect of miR-7 and its role in the prevention and treatment of rotavirus-induced diarrhea, we developed a rotavirus diarrhea model in suckling mice. We synthesized chemically modified reagents for animal experiments, including the agonist miR-7 agomir, antagonist miR-7 antagomir, and negative control (negative oligonucleotide control), and administered them orally to 4-day-old suckling mice. Twenty-four hours after inoculation, suckling mice were infected with 10 ID50 of rotavirus and monitored for diarrhea.

**Figure 7:**
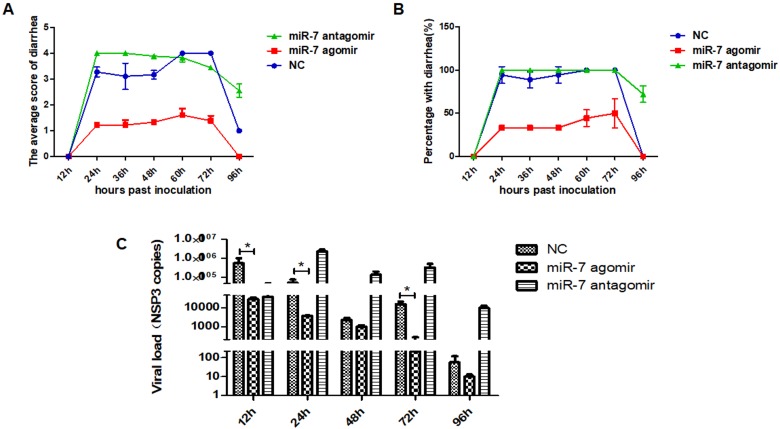
MicroRNA-7 inhibits rotavirus genome replication in vivo. (**A**) The statistical results of the average diarrhea scores of the sulking mice from 12 hpi to 96 hpi. Mean diarrhea score: the sum of all diarrhea or not-diarrhea scores each group/n (n means three experiments, *n* = 3). (**B**) The percentage of diarrhea of the sulking mice from 12 hpi to 96 hpi. Percentage with diarrhea (%): the number of diarrhea mice/the total number of mice in this group. (**C**) Viral gene (*NSP3*) copies detection in jejunal tissue of neonatal mice by RT-PCR from 12 hpi to 96 hpi. RNA of jejunal tissue was extracted for RT-PCR assay. Data are expressed as the mean ±SD, * *p* < 0.01.

The diarrhea symptoms in suckling mice were scored according to Boshuizen’s grading criteria [47,48]. The results showed that 24 h after rotavirus infection, diarrhea symptoms were serious (both with a score of 4 points) in both the NC and miR-7 antagomir group and begin recovery at 96 hpi, while no significant diarrhea symptoms were observed in the miR-7 agomir group. In addition, the diarrhea symptoms were more severe in the miR-7 antagomir group than in the NC group (Figure 7A,B). The suckling mice were euthanized at 12, 24, 48, 72 and 96 hpi, and intestinal tissues were harvested. Total RNA was extracted after the tissue was homogenized by grinding the tissue while frozen. Using NSP3 primers, the rotavirus copy number in the intestinal tissue was analyzed by qRT-PCR. The results showed that, comparing with NC group, the rotavirus copy number was reduced in the miR-7 agomir group and increased in the miR-7 antagomir group. (Figure 7C).

**Figure 8:**
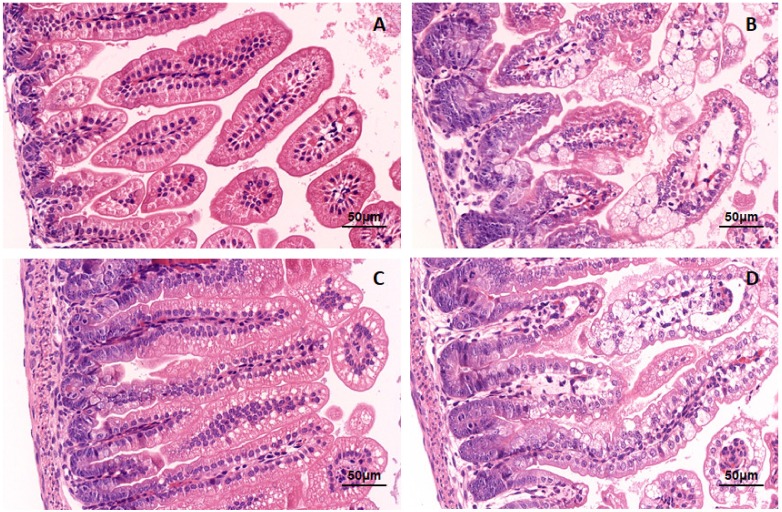
Histopathological changes in the suckling mice infected with rotavirus. The 4-day-old sucking mice were administered with the agonist miR-7 agomir (5 nmol per mouse), antagonist miR-7 antagomir (10 nmol per mouse), and negative control (5 nmol per mouse) for 24h before RV infection. Then The mice were orally inoculated using oral needle with 10^5^ plaque-forming units (PFU) of rotavirus (10 ID50). (**A**) Mock group, the jejunum of neonatal mice infected with medium. (**B**) NC group, the jejunum of neonatal mice administered with negative control oligonucleotides (NC). (**C**) miR-7 agomir group, the jejunum of neonatal mice administered with miR-7 agomir. (**D**) miR-7 antagomir group, the jejunum of neonatal mice administered with miR-7 antagomir.

**Figure 9:**
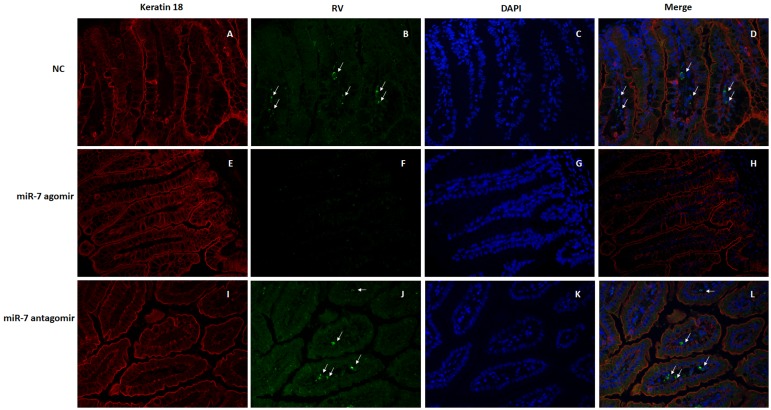
Immunofluorescence detection of RV antigen in the jejunum of the neonatal mice inoculated with rotavirus. The 4-day-old sucking mice were administered with the agonist miR-7 agomir (5 nmol per mouse), antagonist miR-7 antagomir (10 nmol per mouse), and negative control (5 nmol per mouse) for 24h before RV infection. Then the mice were orally inoculated using oral needle with 10^5^ plaque-forming units (PFU) of rotavirus (10 ID50). The jejunum paraffin sections of infected mice were assayed using Goat anti rotaviral antibody and mouse anti keratin 18. Red fluorescence represents keratin 18, green fluorescence represents RV antigen, blue fluorescence represents DAPI. (**A**–**D**) NC group, the jejunum of neonatal mice administered with negative control oligonucleotides (NC). (**E**–**H**) miR-7 agomir group, the jejunum of neonatal mice administered with miR-7 agomir. (**I**–**L**) miR-7 antagomir group, the jejunum of neonatal mice administered with miR-7 antagomir.

In order to observe the pathological changes within the intestine, intestinal tissue was fixed and paraffin-embedded for analysis via H&E staining. The small intestinal villi of NC group mice displayed obvious swelling and damage, in addition to a large number of visible vacuolated cells at the tip of the small intestine villi. The pathological changes were more severe in the miR-7 antagomir group than in the NC group, which exhibited more obvious swelling and damage of the intestinal villi and a larger number of vacuolated cells. However, in the miR-7 agomir group, the small intestinal villi of the suckling mice only showed slight swelling and immune cell infiltration; no damage was observed (Figure 8). Meanwhile, the jejunum paraffin sections of infected mice were assayed using Goat anti rotaviral antibody and mouse anti keratin 18 by immunofluorescence to detect RV replication in jejunum. Rotavirus-specific fluorescence can be detected in the small intestine of mice in the NC group and antagomir group (Shown by arrow) (Figure 9A–D,I–L). However, in the miR-7 agomir group, no significant specific fluorescence can be observed (Figure 9E–H).

**Figure 10:**
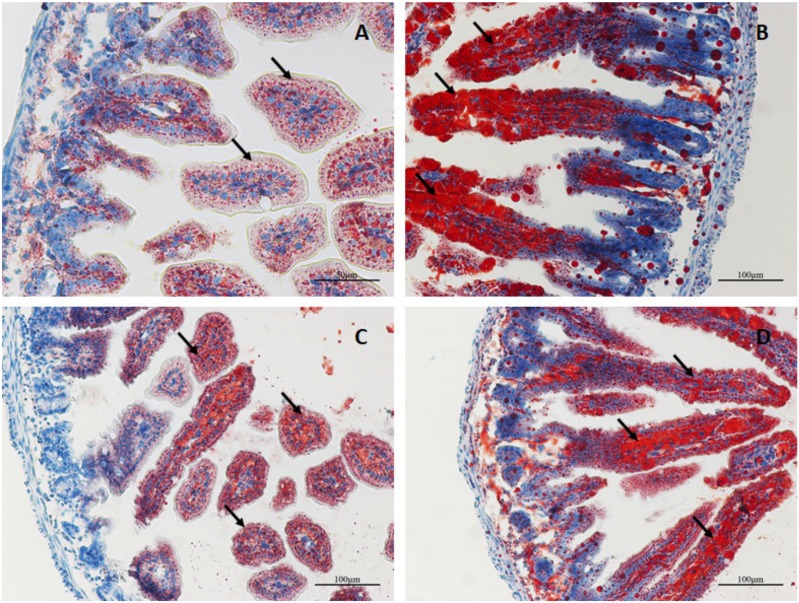
Oil red O staining of the jejunum of the neonatal mice inoculated with rotavirus. (**A**) Mock group, the jejunum of neonatal mice infected with medium. (**B**) NC group, the jejunum of neonatal mice administered with negative control oligonucleotides (NC). (**C**) miR-7 agomir group, the jejunum of neonatal mice administered with miR-7 agomir. (**D**) miR-7 antagomir group, the jejunum of neonatal mice administered with miR-7 antagomir. Red represents lipid droplets stained with oil red O.

After infection, rotavirus recruits a large amount of lipid droplets for virus replication. In order to visualize the level of lipid droplet formation in the suckling mice, cryosections of the intestinal tissue were prepared for oil red O staining. The results showed an increase in the amount of lipid droplets that formed and aggregated in the intestinal tissue of the infected mice. However, lipid droplet formation and aggregation were significantly lower in the miR-7 agomir group. There was no significant difference in lipid formation or aggregation between miR-7 agomir and NC groups (Figure 10).

## 4. Discussion

With the in-depth exploration of the RNA field, the role of non-coding RNAs in numerous biological processes has gradually been revealed. Among them, miRNAs and siRNAs play an important role in many biological processes because they regulate the post-transcriptional expression of genes. MiRNAs bind to the complementary sequence of the target mRNA, thereby triggering degradation or inhibiting translational of the target mRNA. This type of regulation may lead to a novel manner of inhibiting the expression of pathogenic genes that could potentially be used in small RNA drug development. MIR-7 was first discovered as a tumor suppressor, which achieved anti-tumor effects by directly targeting tumor growth-promoting genes or modifying regulatory tumor suppressor factors [49,50,51,52,53,54]. In addition to anti-tumor effects, miR-7 also undergoes expression changes after viral infection and plays a role in antiviral defense by regulating gene expression and related pathways in host cells. Studies have found that oncolytic adenovirus can exert anti-tumor effects through autophagic cell death induced by the E2F1-MIR-7 epidermal growth factor receptor (EGFR) pathway [55]. Upregulation of miR-7 expression by the hepatitis B virus (HBV)-encoded HBx protein inhibits EGFR expression and controls the growth rate of hepatocellular carcinoma (HCC) cells [56].

Rotavirus is one of the main causes of diarrhea in infants and young children under five years of age. There is currently no effective targeted therapy available. Vaccines are an effective way of preventing rotavirus infectious. There are few reports on the role of host microRNAs in antiviral defense and less is known about their mechanisms. In previous study [39], wild-type rotavirus ZTR-68 strain was isolated and used to infect the sensitive cell line MA104. Using deep RNA sequencing, the microRNA expression profile during rotavirus infection was analyzed. We found that miR-7 was upregulated nearly 420-fold after RV infection. In this study, these findings were further confirmed by qRT-PCR, which showed an approximate 20-fold increase in the expression of miR-7. The expression of miR-7 increased as the infection dose of rotavirus increased, indicating that miR-7 expression level is closely related to rotavirus concentration. Upregulation expression of miR-7 can be detected after different G-type rotaviruses infections. This up-regulation response may be one of the strategies for host cells to fight rotaviruses infection.

After transfecting the miR-7 mimic, the expression of miR-7 was upregulated in MA104 cells. Compared with the control group, the expression of rotavirus protein was lower in host cells with miR-7 upregulation at 24 hpi. These results suggest that miR-7 might play a negative regulatory role in virus proliferation and may have antiviral effects. Target prediction results showed that miR-7 can bind on the *NSP5* gene of different genotype rotavirus. Interestingly, we once adaptive cultured ZTR-68 strain rotavirus from MA104 cells to Vero cells. Adaptive culture of the same strain in different host cells didn’t change the binding site of miR-7 on the *NSP5* gene (Figure 4F). MiR-7 regulates *NSP5* expression is a stable regulation strategy in virus adaptive culture.

NSP5 protein is encoded by the 11th gene of the rotavirus RNA genome and is capable of binding to RNA, interacting with NSP2, and promoting viroplasm formation and viral replication cycles. Through a series of experiments, we found, for the first time, that the gene that encodes NSP5 is a new target gene of miR-7, and that miR-7 can inhibit the expression of NSP5 protein by binding to its gene. Using transmission electron microscopy, we observed a decrease in the number of viroplasms in cells with upregulated miR-7, indicating that miR-7 influenced the formation of viroplasm during virus replication. After serial passaging rotavirus for three generations in MA104 cells expressing upregulated miR-7, the titer of progeny virus was lower compared with the control group. The viroplasm is the location where replication of the rotavirus genome occurs. By inhibiting the expression of the NSP5 protein, miR-7 causes a reduction in viroplasm and, therefore, the replication and titer of progeny rotavirus.

The rotavirus-induced diarrhea model is an important way to evaluate the effectiveness of a rotavirus vaccine or viral infectivity [56]. In the current study, we established a rotavirus diarrhea model in suckling mice. After administration of miR-7 agomir, mice were infected with rotavirus. Results indicated that oral introduction of miR-7 significantly alleviated, even prevented, rotavirus-induced diarrhea in suckling mice. Overexpression of miR-7 can reducing diarrhea by inhibiting rotavirus replication in the intestinal of sulking mice. Antiviral therapy for rotavirus infection has been studied but remains mostly in preclinical stages. RNA interference technology has been widely used in various antiviral research, such as HIV-1, hepatitis C virus (HCV), and influenza viruses. To date, there are limited studies regarding the use of small RNA molecules in anti-rotavirus infection. MiR-7 has the potential to become an anti-rotavirus small RNA drug, and the MiR-7-NSP5 binding site could be used as a therapeutic target for novel and effective anti-rotavirus treatments.

## 5. Conclusions

In the present study, we found that miR-7 expression levels were increased significantly following rotavirus infection. Overexpression of miR-7 inhibited rotavirus replication in vitro. The rotavirus gene that encodes the NSP5 protein was predicted and further confirmed to be the target gene of miR-7. Through NSP5, miR-7 altered viroplasm formation and virus genome replication and further influenced the titer of progeny virus. Oral administration of the small molecule mimic miR-7 agomir significantly inhibited rotavirus-induced diarrhea in suckling mice, further indicating the suppressive function of miR-7 on rotavirus replication.

## References

[1] Tate J.E., Burton A.H., Boschi-Pinto C., Parashar U.D., Agocs M., Serhan F., de Oliveira L., Mwenda J.M., Mihigo R., For the World Health Organization–Coordinated Global Rotavirus Surveillance Network (2016). Global, Regional, and National Estimates of Rotavirus Mortality in Children <5 Years of Age, 2000–2013. Clin. Infect. Dis. Off. Publ. Infect. Dis. Soc. Am..

[2] Matthijnssens J., Bilcke J., Ciarlet M., Martella V., Banyai K., Rahman M., Zeller M., Beutels P., Van Damme P., Van Ranst M. (2009). Rotavirus disease and vaccination: Impact on genotype diversity. Future Microbiol..

[3] Estes M.K., Howley P.M. (2013). Fields Virology.

[4] Long C.P., McDonald S.M. (2017). Rotavirus genome replication: Some assembly required. PLoS Pathog..

[5] Eichwald C., Vascotto F., Fabbretti E., Burrone O.R. (2002). Rotavirus NSP5: Mapping phosphorylation sites and kinase activation and viroplasm localization domains. J. Virol..

[6] Afrikanova I., Fabbretti E., Miozzo M.C., Burrone O.R. (1998). Rotavirus NSP5 phosphorylation is up-regulated by interaction with NSP2. J. Gen. Virol..

[7] Martin D., Ouldali M., Menetrey J., Poncet D. (2011). Structural organisation of the rotavirus nonstructural protein NSP5. J. Mol. Biol..

[8] Sen A., Sen N., Mackow E.R. (2007). The formation of viroplasm-like structures by the rotavirus NSP5 protein is calcium regulated and directed by a C-terminal helical domain. J. Virol..

[9] Sotelo P.H., Schumann M., Krause E., Chnaiderman J. (2010). Analysis of rotavirus non-structural protein NSP5 by mass spectrometry reveals a complex phosphorylation pattern. Virus Res..

[10] Sen A., Agresti D., Mackow E.R. (2006). Hyperphosphorylation of the rotavirus NSP5 protein is independent of serine 67, [corrected] NSP2, or [corrected] the intrinsic insolubility of NSP5 is regulated by cellular phosphatases. J. Virol..

[11] Criglar J.M., Hu L., Crawford S.E., Hyser J.M., Broughman J.R., Prasad B.V., Estes M.K. (2014). A novel form of rotavirus NSP2 and phosphorylation-dependent NSP2-NSP5 interactions are associated with viroplasm assembly. J. Virol..

[12] Contin R., Arnoldi F., Campagna M., Burrone O.R. (2010). Rotavirus NSP5 orchestrates recruitment of viroplasmic proteins. J. Gen. Virol..

[13] Eichwald C., Rodriguez J.F., Burrone O.R. (2004). Characterization of rotavirus NSP2/NSP5 interactions and the dynamics of viroplasm formation. J. Gen. Virol..

[14] Lopez T., Rojas M., Ayala-Breton C., Lopez S., Arias C.F. (2005). Reduced expression of the rotavirus NSP5 gene has a pleiotropic effect on virus replication. J. Gen. Virol..

[15] Martin D., Charpilienne A., Parent A., Boussac A., D’Autreaux B., Poupon J., Poncet D. (2013). The rotavirus nonstructural protein NSP5 coordinates a [2Fe-2S] iron-sulfur cluster that modulates interaction to RNA. FASEB J..

[16] Campagna M., Eichwald C., Vascotto F., Burrone O.R. (2005). RNA interference of rotavirus segment 11 mRNA reveals the essential role of NSP5 in the virus replicative cycle. J. Gen. Virol..

[17] Chnaiderman J., Barro M., Spencer E. (2002). NSP5 phosphorylation regulates the fate of viral mRNA in rotavirus infected cells. Arch. Virol..

[18] Schirle N.T., Sheu-Gruttadauria J., MacRae I.J. (2014). Structural basis for microRNA targeting. Science.

[19] Lim L.P., Glasner M.E., Yekta S., Burge C.B., Bartel D.P. (2003). Vertebrate microRNA genes. Science.

[20] Seeley J.J., Baker R.G., Mohamed G., Bruns T., Hayden M.S., Deshmukh S.D., Freedberg D.E., Ghosh S. (2018). Induction of innate immune memory via microRNA targeting of chromatin remodelling factors. Nature.

[21] Zhang X., Zuo X., Yang B., Li Z., Xue Y., Zhou Y., Huang J., Zhao X., Zhou J., Yan Y. (2014). MicroRNA directly enhances mitochondrial translation during muscle differentiation. Cell.

[22] Xiao C., Rajewsky K. (2009). MicroRNA control in the immune system: Basic principles. Cell.

[23] van Rooij E., Sutherland L.B., Qi X., Richardson J.A., Hill J., Olson E.N. (2007). Control of stress-dependent cardiac growth and gene expression by a microRNA. Science.

[24] Abdul-Careem M.F., Hunter B.D., Sarson A.J., Parvizi P., Haghighi H.R., Read L., Heidari M., Sharif S. (2008). Host responses are induced in feathers of chickens infected with Marek’s disease virus. Virology.

[25] Zuo L., Yue W., Du S., Xin S., Zhang J., Liu L., Li G., Lu J. (2017). An update: Epstein-Barr virus and immune evasion via microRNA regulation. Virol. Sin..

[26] Huang T., Zhang X. (2012). Functional analysis of a crustacean microRNA in host-virus interactions. J. Virol..

[27] Qi L., Wang K., Chen H., Liu X., Lv J., Hou S., Zhang Y., Sun Y. (2019). Host microRNA miR-1307 suppresses foot-and-mouth disease virus replication by promoting VP3 degradation and enhancing innate immune response. Virology.

[28] Cazalla D., Yario T., Steitz J.A. (2010). Down-regulation of a host microRNA by a Herpesvirus saimiri noncoding RNA. Science.

[29] Aparicio R., Simoes Da Silva C.J., Busturia A. (2015). MicroRNA miR-7 contributes to the control of Drosophila wing growth. Dev. Dyn. Off. Publ. Am. Assoc. Anat..

[30] Correa-Medina M., Bravo-Egana V., Rosero S., Ricordi C., Edlund H., Diez J., Pastori R.L. (2009). MicroRNA miR-7 is preferentially expressed in endocrine cells of the developing and adult human pancreas. Gene Expr. Patterns GEP.

[31] Lopez-Beas J., Capilla-Gonzalez V., Aguilera Y., Mellado N., Lachaud C.C., Martin F., Smani T., Soria B., Hmadcha A. (2018). miR-7 Modulates hESC Differentiation into Insulin-Producing Beta-like Cells and Contributes to Cell Maturation. Mol. Ther. Nucleic Acids.

[32] (2018). Erratum: CircRNA CDR1as/miR-7 signals promote tumor growth of osteosarcoma with a potential therapeutic and diagnostic value [Corrigendum]. Cancer Manag. Res..

[33] Akalay I., Tan T.Z., Kumar P., Janji B., Mami-Chouaib F., Charpy C., Vielh P., Larsen A.K., Thiery J.P., Sabbah M. (2015). Targeting WNT1-inducible signaling pathway protein 2 alters human breast cancer cell susceptibility to specific lysis through regulation of KLF-4 and miR-7 expression. Oncogene.

[34] Bhere D., Tamura K., Wakimoto H., Choi S.H., Purow B., Debatisse J., Shah K. (2018). microRNA-7 upregulates death receptor 5 and primes resistant brain tumors to caspase-mediated apoptosis. Neuro-Oncology.

[35] Wan S., Wang J., Wang J., Wu J., Song J., Zhang C.Y., Zhang C., Wang C., Wang J.J. (2017). Increased serum miR-7 is a promising biomarker for type 2 diabetes mellitus and its microvascular complications. Diabetes Res. Clin. Pract..

[36] Zhang X., Liu D., Zhang S., Wei X., Song J., Zhang Y., Jin M., Shen Z., Wang X., Feng Z. (2015). Host-virus interaction: The antiviral defense function of small interfering RNAs can be enhanced by host microRNA-7 in vitro. Sci. Rep..

[37] Saleh M.C., Van Rij R.P., Andino R. (2004). RNA silencing in viral infections: Insights from poliovirus. Virus Res..

[38] Huang Y., Wang W., Xu Z., Pan J., Zhao Z., Ren Q. (2018). Eriocheir sinensis microRNA-7 targets crab Myd88 to enhance white spot syndrome virus replication. Fish. Shellfish Immunol..

[39] Zhou Y., Wu J., Geng P., Kui X., Xie Y., Zhang L., Liu Y., Yin N., Zhang G., Yi S. (2016). MicroRNA profile analysis of host cells before and after wild human rotavirus infection. J. Med. Virol..

[40] Li S., Zhang G., Wu J., Yin N., Yi S., Mi K., Cao Y., Li Y., Sun M., Li H. (2013). Isolation human rotavirus strains and adaptive culture. China Biotechnol..

[41] Enright A.J., John B., Gaul U., Tuschl T., Sander C., Marks D.S. (2003). MicroRNA targets in Drosophila. Genome Biol..

[42] John B., Enright A.J., Aravin A., Tuschl T., Sander C., Marks D.S. (2004). Human MicroRNA targets. PLoS Biol..

[43] Zhou Y., Geng P., Liu Y., Wu J., Qiao H., Xie Y., Yin N., Chen L., Lin X., Liu Y. (2018). Rotavirus-encoded virus-like small RNA triggers autophagy by targeting IGF1R via the PI3K/Akt/mTOR pathway. Biochim. Biophys. Acta Mol. Basis Dis..

[44] Zhou Y., Qiao H., Yin N., Chen L., Xie Y., Wu J., Du J., Lin X., Wang Y., Liu Y. (2019). Immune and cytokine/chemokine responses of PBMCs in rotavirus-infected rhesus infants and their significance in viral pathogenesis. J. Med. Virol..

[45] Gil M.T., de Souza C.O., Asensi M., Buesa J. (2000). Homotypic protection against rotavirus-induced diarrhea in infant mice breast-fed by dams immunized with the recombinant VP8* subunit of the VP4 capsid protein. Viral Immunol..

[46] Offit P.A., Clark H.F., Kornstein M.J., Plotkin S.A. (1984). A murine model for oral infection with a primate rotavirus (simian SA11). J. Virol..

[47] Li J.T., Wei J., Guo H.X., Han J.B., Ye N., He H.Y., Yu T.T., Wu Y.Z. (2016). Development of a human rotavirus induced diarrhea model in Chinese mini-pigs. World J. Gastroenterol..

[48] Boshuizen J.A., Reimerink J.H., Korteland-van Male A.M., van Ham V.J., Koopmans M.P., Buller H.A., Dekker J., Einerhand A.W. (2003). Changes in small intestinal homeostasis, morphology, and gene expression during rotavirus infection of infant mice. J. Virol..

[49] Li Q., Zhu F., Chen P. (2012). miR-7 and miR-218 epigenetically control tumor suppressor genes RASSF1A and Claudin-6 by targeting HoxB3 in breast cancer. Biochem. Biophys. Res. Commun..

[50] Chakrabarti M., Ai W., Banik N.L., Ray S.K. (2013). Overexpression of miR-7-1 increases efficacy of green tea polyphenols for induction of apoptosis in human malignant neuroblastoma SH-SY5Y and SK-N-DZ cells. Neurochem. Res..

[51] Hansen T.B., Kjems J., Damgaard C.K. (2013). Circular RNA and miR-7 in cancer. Cancer Res..

[52] Xie J., Chen M., Zhou J., Mo M.S., Zhu L.H., Liu Y.P., Gui Q.J., Zhang L., Li G.Q. (2014). miR-7 inhibits the invasion and metastasis of gastric cancer cells by suppressing epidermal growth factor receptor expression. Oncol. Rep..

[53] Zhang H., Cai K., Wang J., Wang X., Cheng K., Shi F., Jiang L., Zhang Y., Dou J. (2014). MiR-7, inhibited indirectly by lincRNA HOTAIR, directly inhibits SETDB1 and reverses the EMT of breast cancer stem cells by downregulating the STAT3 pathway. Stem. Cells.

[54] Yin C.Y., Kong W., Jiang J., Xu H., Zhao W. (2019). miR-7-5p inhibits cell migration and invasion in glioblastoma through targeting SATB1. Oncol. Lett..

[55] Tazawa H., Yano S., Yoshida R., Yamasaki Y., Sasaki T., Hashimoto Y., Kuroda S., Ouchi M., Onishi T., Uno F. (2012). Genetically engineered oncolytic adenovirus induces autophagic cell death through an E2F1-microRNA-7-epidermal growth factor receptor axis. Int. J. Cancer.

[56] Chen Y.J., Chien P.H., Chen W.S., Chien Y.F., Hsu Y.Y., Wang L.Y., Chen J.Y., Lin C.W., Huang T.C., Yu Y.L. (2013). Hepatitis B Virus-Encoded X Protein Downregulates EGFR Expression via Inducing MicroRNA-7 in Hepatocellular Carcinoma Cells. Evid. Based Complement. Altern. Med. eCAM.

